# Stereopsis in Sports: Visual Skills and Visuomotor Integration Models in Professional and Non-Professional Athletes

**DOI:** 10.3390/ijerph182111281

**Published:** 2021-10-27

**Authors:** Valentina Presta, Costanza Vitale, Luca Ambrosini, Giuliana Gobbi

**Affiliations:** 1 Department of Medicine & Surgery (DiMeC), University of Parma, 43126 Parma, Italy; valentina.presta@unipr.it (V.P.); luca.ambrosini@unipr.it (L.A.); 2 TSRM-PSTRP National Federation, 43126 Parma, Italy; costanza.vitale1997@gmail.com

**Keywords:** depth perception, sport vision, sport expertise, athletic performance

## Abstract

Visual skills in sport are considered relevant variables of athletic performance. However, data on the specific contribution of stereopsis—as the ability to perceive depth—in sport performance are still scarce and scattered in the literature. The aim of this review is therefore to take stock of the effects of stereopsis on the athletic performance, also looking at the training tools to improve visual abilities and potential differences in the visuomotor integration processes of professional and non-professional athletes. Dynamic stereopsis is mainly involved in catching or interceptive actions of ball sports, whereas strategic sports use different visual skills (peripheral and spatial vision) due to the sport-specific requirements. As expected, professional athletes show better visual skills as compared to non-professionals. However, both non-professional and professional athletes should train their visual skills by using sensory stations and light boards systems. Non-professional athletes use the visual inputs as the main method for programming motor gestures. In contrast, professional athletes integrate visual information with sport expertise, thus, they encode the match (or the athletic performance) through a more complex visuomotor integration system. Although studies on visual skills and stereopsis in sports still appear to be in their early stages, they show a large potential for both scientific knowledge and technical development.

## 1. Introduction

Visual skills in sport have emerged as relevant variables of athletic performance. In the early 1980s, Stine et al. showed that athletes had better visual abilities than non-athletes, and that, among athletes, there were differences between those with poorer and those with better visual skills. It was hypothesised that certain visual skills were optimal for selected sports, and the trainability of such visual abilities was proposed; however, the relationship between enhanced visual skills and the improvement of athletic performance remained unclear [1,2]. Independently from the sport played, athletes constantly stimulate the vision system as it contributes to the gesture planning. The main visual abilities involved in the sport vision field are: eye–limb coordination, static and dynamic acuity, peripheral vision, spatial focus, speed and distance of subjects and objects in the environment [3]. Among these, stereopsis—namely, the perception of depth [4]—is essential in the three-dimensional analysis of the environment. It is, therefore, necessary to the rapid and precise evaluation of spatial movements and distance between athletes and objects, as established by Fitts’ law, for which the time to reach an objective (an area, a button) in a targeting task depends on the distance to the object to be reached and on the size of that object [5]. Technically, stereopsis, calculated in seconds of arc, represents the minimum distance at which it is possible to perceive depth. Therefore, the greater the value of the minimum distance, the greater the stereopsis and the perception of depth of subjects/objects [6].

A possible correlation between stereopsis and sport was previously investigated in catching performances. First, Lenoir et al. (1999) [7] showed that the performance of catchers with low stereopsis decreased as the ball speed increased (athletes made more temporal errors), suggesting a relationship between high-level stereopsis and the ability to catch the ball. This result was more recently confirmed by Mazyn et al. (2004; 2007) [8,9]. They also reported a significant effect of specific vision training in catchers with both higher and lower stereopsis, although athletes with a low stereopsis showed only moderate improvements. Catching performances are partly representative of the variety of sport situations in which stereopsis can be involved: for example, the interceptive actions typical of catching performances are specific to racket sports (e.g., tennis, badminton) and to some team sports with hitters and pitchers, such as baseball and cricket. Team sports with strategy elements (e.g., basketball, volleyball, hockey, soccer) are more complex, and the need for visual skills is related to the position, opponents’ actions, ball position and movement, etc. [10,11]. In these sports, dynamic stereopsis is primarily involved to perceive the depth while following subjects/objects. On the contrary, static stereopsis—depth perception of static objects—is required in sports such as golf or archery, in which the target is fixed [11].

There is evidence that visual skills can differ between athletes and non-athletes, experts versus novices, and ball versus non-ball players, demonstrating how visual abilities influence the athletic performance just as the motor skills do. For example, Boden et al. (2009) [11] compared data from tests of depth perception in baseball/softball players (10–18 years old) versus non-ball, age-matched players, reporting superior abilities in ball players. Similarly, basketball players aged 11–13 showed remarkable visual abilities (three-dimensional vision, distance visual acuity, visual reaction time, etc.), comparable to adults or even higher than reference values [12]. However, it is not clear whether the best levels of visual abilities, such as stereopsis, are conditional to the best performance, or not. Whether the optimal athletic performance depends on the ability to use the relevant visual information, or whether it is the sport expertise that guides the best visual encoding of a performance, has also been discussed.

Therefore, the aim of this narrative review is to summarise the existing evidence on the influence of stereopsis on sport performances, in order to provide practical recommendations for athletes, coaches and trainers. In particular, the review is centred on the: (a) integration among visual skills with optimal sport performances; (b) the approach for the evaluation of stereopsis in sport; (c) the comparisons between athletes and non-athletes; and (d) training strategies for the improvement of visual skills.

## 2. Stereopsis and Sports

Stereopsis is one of the necessary visual skills used in daily life, including reading, writing, walking, driving, physical activity, etc. and its impairment negatively affects interactions with subjects or objects [13,14]. On the contrary, normal, or higher levels of stereopsis support the performance of many tasks, including motor gestures. The type of sport and the position played, as well as the setting and modality of unfolding performance, altogether define the set of the visual abilities required and the consequent relevance of stereopsis [14]. It is, in fact, conceivable that stereopsis is more relevant in some sport disciplines than in others, in which the contribution of such visual ability is lower and/or complementary to other visual (and/or non-visual) skills.

### 2.1. Interceptive Sports

**Figure 1:**
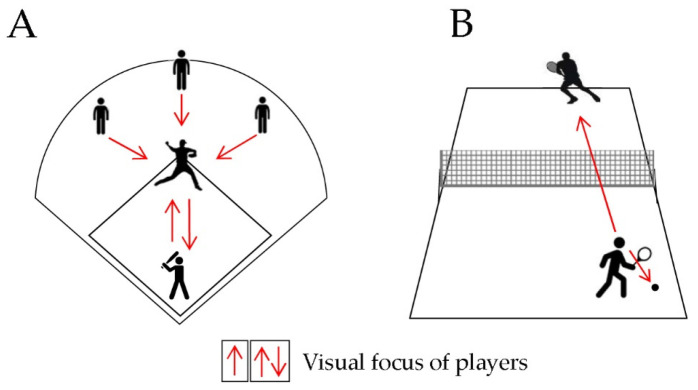
Representative models of baseball (**A**) and tennis (**B**) fields, players, and match development. The arrows indicate the visual burden: (**A**) the two-way arrows between the pitcher and the batter show the mutual visual focus before and during the throw action of pitcher; the outfield players wait for the throw development (arrows directed from outfield players to the pitcher); (**B**) the visual burden is limited to the ball and the opponent’s movements (both arrows from the player directed to the ball and the opponent).

Catching performances were investigated first. Catching and pitching are the basic gestures of many sport disciplines, being critical in sports such as baseball and cricket. In this case, athletes must follow the moving object, while coordinating to hit or catch it at the right time, evaluating speed, trajectory, and others moving in the field. In this complex situation, a sum of visual cues must integrate the motor programming. Although depth perception is not the only visual skill useful for interceptive actions, it is sufficient to discriminate athletes from non-athletes, because athletes have a significantly higher capacity to integrate visual and visuomotor skills to control the interceptive action [15]. The correlation between visual cues and motor responses is evident in cricket and baseball. Although the sports are different in terms of the game development and equipment (i.e., whether the bat has a circular (baseball) or a flat surface (cricket)), the players have similar roles. Firstly, the visual focus of pitcher and catcher is reciprocally directed to each other; then, in the early phases of the ball pitch the visual skills are essential to predict the ball speed, trajectory and when the action of hitting the ball must start (Figure 1A). Figure 1 represents typical match situations in which teammates and opponents are involved. For example, a baseball match (panel A) requires a first focus on the visual relationship between the batter and the pitcher (two-way arrows), together with the other players, looking at the action development (the arrows between outfield players and the pitcher). When the pitcher throws the ball, the visual performance of all players changes in function of ball trajectory, requiring depth perception, fixation, and following-the-object skills.

Another typical situation of a tennis match is depicted in panel B of Figure 1: the visual burden of player with ball possession is mainly focused on ball catching performance (the arrow directed to the ball) and movement of the opponent (the arrow directed towards the opponent).

The reaction time of batsmen needs to be very short as the ball’s flight is fast (0.38 s in Regan’s study, 2012) [16]. On the other hand, catchers in the field can move only after the ball is hit and, as batsmen, they constantly recall the visual information to analyse the ball trajectory, while running in the predicted catching point. Interestingly, the relationship between the pitcher and batsmen revolves around the perception of ball direction and motion in depth; namely, the pitcher avoids giving visual cues to the batsmen when he tries to encode his future throw. On the other hand, the batsman focuses on the first moments of the pitch to evaluate the ball trajectory and start the hitting action [17].

Depth perception is made possible by the integration of right- and left-eye information (binocular vision); therefore, one might expect that loss of one eye vision could negatively affect the performance. This is partly true as evidence [16] has reported that monocular vision has little impact on batting performance, showing, however, that losing the left eye for a right-handed athlete would have worse consequences. In a comparison among baseball batters, even a temporary vision occlusion did not impact the batting task; that is, athletes with more experience were able to anticipate the specific gesture (downswing and weight transfer) better than less skilled athletes [18]. Therefore, athletes have better visual abilities than to non-athletes, and among athletes the best performance is achieved by athletes trained in his sport-specific visual demand [19,20].

Interceptive actions characterise ball sports, but also racquet sports, such as tennis (Figure 1B) or badminton, and they essentially require the same visual abilities when analysing the ball trajectories. Studies on badminton players [19,21] were consistent with the above-mentioned differences between athletes and non-athletes. Interestingly, the authors linked the superior visuomotor performance of athletes to a faster transformation at cortical level of visual cues in motor responses, suggesting that athletes were able to encode the performance by shifting rapidly from visual signals into motor actions [19].

#### Stereopsis in Putting and Shooting Actions

Interceptive actions identify a group of disciplines, mainly ball sports, in which the athlete intercepts the ball while it is moving. However, there are ball sports, such as golf, in which, although the ball is stationary and near the player, stereopsis is still involved (static stereopsis). Recent studies showed that expert players performed much better than beginners in: (i) evaluating the distance between the ball and the hole [22]; (ii) the putting dynamics; (iii) the prediction of putting success [23,24]. More specifically, an intriguing study of Micarelli et al. (2019) [25] showed a vision-independent behaviour of expert golfers compared to non-experts during swing performance. When focusing on body movement, expert golfers base their motor control on proprioceptive and vestibular signals rather than visual inputs, which they instead integrate in the swing motor programming. Similarly, Kim et al. (2019) [26] analysed the differences in shooting behaviour of Olympic vs. collegiate archers. Olympic athletes were able to exclude any type of confounding factors during the shot and visual parameters such as fixation or eye movements were optimised as compared to collegiate archers. Although, as said, stereopsis is intrinsically related to binocular vision, it must be observed that: (i) not all archers have both eyes open while shooting, and (ii) due to the long distance from the target, stereopsis cannot be so relevant.

Although stereopsis is considered an important skill for spatial localisation, studies reported difficulties in evaluating stereopsis with specific tests and at far distances [27,28]. However, a specific vision training method has been proposed to improve putting or shooting skills in both golf and archery. Overall, it appears that in putting/shooting sport actions, non-professional athletes base their gesture programming mainly on stereoptic inputs, which, however, should be—with experience—progressively integrated by attentional, proprioceptive, and vestibular inputs [22,26].

### 2.2. Strategic Sports

**Figure 2:**
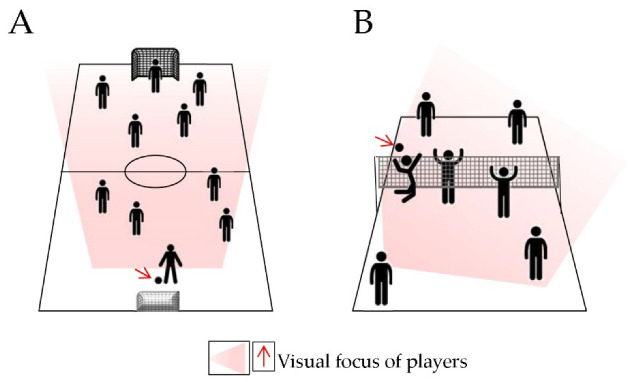
Representative models of soccer (**A**) and volleyball (**B**) fields, players, and game development. The blurred ray panels (**A**,**B**) show the visual focus (vison in depth) of players; the red arrows show the visual focus of strikers on the ball.

Strategy components emerge in team sports, such as basketball, football or soccer, hockey, volleyball. These are all ball sports, but the presence of teammates and opponents further engages the visual system, requiring the integration of ball and subjects positioning all along the match to support the decision-making processes of each athlete [29] (Figure 2A,B).

Comparisons between basketball players and non-athletes showed differences in several visual skills [30]. Athletes had better scores in tracking objects tasks (dynamic vision attention), particularly at higher speeds. Performance was improved in athletes with high levels of visual function, as they were able to steal the ball or to assist teammates successfully [31]. The relevance of visual inputs was also studied during free throwing in basketball: players were asked to throw at increasing distances from the basket [32] and in a physiological or blurred vision condition [33]. As expected, performance was better in physiological vision conditions and at more practiced distances, but at far distances and in blurred conditions, the expertise of the players was not sufficient to compensate for the lack of visual information to obtain the expected performance, suggesting the paramount role of visual inputs for this basketball skill [33].

Experienced soccer players showed differences in many visual skills (peripheral vision, ball and player fixation, quick response time, etc.) compared to inexperienced subjects [34,35]. Gaze behaviour of experienced soccer players exhibited a faster adaptation while focusing on a larger field area, better performing the so-called “zooming-out” operations as compared to non-athletes or novices [36]. Similarly, Basevitch et al. (2015) [37] investigated passing and target distance estimation tasks in low- and high-skilled soccer players in different conditions (physiological, occluded, or distorted vision). The results of this study demonstrated better performances of high-skilled players, showing that visual information was essential for the quality of motor tasks independently from the skill level of participants. Stereopsis did not differ between expert and amateur soccer players, although athletes showed better reaction times in the monocular test (faster recognition of an object) [38]. As for the decision-making process during a match, results from soccer referee’s visual skills were compared and it was possible to match the best scores on visual tests (visual memory, recognition speed, peripheral vision, etc.) with the best refereeing performance (i.e., with fewer errors in penalties awarded or offside decisions) [39]. A similar trend has been reported in volleyball players who showed better accuracy in predicting the ball direction and spent less time in ball fixation during their field search strategy as compared to novice athletes [40]. Visual abilities in team sports can be different according to the field measures and the playing position. Although in general strikers and goalkeepers show the best selected visual skills, to the best of our knowledge, position-related definitive data are not available yet. A single study on hockey did not show differences in visual skills among players, independent of their position [41]. On the contrary, there is evidence on the benefits of vision training when performing in most of the aforementioned disciplines [10,41].

## 3. Vision Training

Previous evidence on the correlation between sport demands and visual skills proposed the concept of vision trainability [2,42]. As previously shown, sport performance also relies on different visual abilities, thus athletes and coaches should plan vision training according to the sport played [43]. In particular, both static and dynamic stereopsis and contrast sensitivity (defined as the ability to detect low-contrast objects of various sizes) [44] were proposed to be the most relevant skills in sport performance, they are also defined as sport-related abilities and are better developed among athletes as compared to non-athletes [38,44,45].

Various training procedures of visual skills have been described, with different training programs according to the different disciplines. For example, Zwierko et al. (2015) [43] proposed a set of orthoptic exercises to improve binocular vision in soccer, basketball, and handball athletes with positive effects on visual function after an 8-week training program. Specifically, after a preliminary ocular warm-up, the training consisted of saccadic and pursuit eye movements, fixation and dynamic vision exercises, convergence and divergence, and relaxing ocular exercises. A different approach was used by Clark et al. (2015) [46] who studied a vision training program as a prevention strategy against sport concussion. The program referred to the standard vision evaluation tests with also a specialised light board training system. Specifically, stereopsis improved after six weeks of training (from 23.7 mm to 36.9 mm), where (collegiate) athletes gained better control and precision of fine eye movements, vergence and fixation patterns. It is of note that the authors reported a detraining effect of stereopsis in the athletes that stopped the vision training. By contrast, Schoemann et al. (2017) [47] reported that stereopsis remained at significant levels even after a 6-month pause in training. This opposite result might be due to the different measurement of stereopsis in the two mentioned studies: in one of them stereopsis was evaluated as “processing time” (the reaction time at which it is possible to perceive depth) [47]; in the other study, it was defined as the minimum distance at which it is possible to perceive depth [46].

Position-specificity of visual training was investigated by Wimshurst et al. (2012) [41]. Hockey players were asked to follow a 10-week computer-based visual training program. The participants were professional athletes (Olympic team) with all different playing positions (forwards, defenders, midfielder, and goalkeepers). Some of the visual tasks proposed were saccadic eye movements, dynamic shape recognition, visual and focus acuity, flexibility, and peripheral vision. In agreement with previous studies, there were no pre-training differences among players. The visual abilities of all players improved after the training program. Interestingly, the goalkeepers outperformed, especially in saccades and focus flexibility. This result suggests that even upon the same visual training program, athletes might preferentially improve their visual skills that are more closely related to their position in the team.

Recently, a specific visual training tool was developed (Nike SPARQ—Speed, Power, Agility, Reaction, and Quickness—Sensory Training Station; Nike Inc., Beaverton, OR, USA). The station digitally evaluates both visual and motor abilities through a battery of nine tasks: visual clarity, near–far quickness, contrast sensitivity, target capture, depth perception, perception span, go/no-go, eye–hand coordination, and response time [48]. Burris et al. (2020) [10] tested a cohort of more than 2000 athletes using this tool: as expected, performance was higher in higher-level athletes as compared to non-professionals, with gender differences in eye–hand reaction times (>females) and near–far eye movements (>males). Of note, differences were found among athletes of interceptive versus strategic sports: baseball and tennis players showed higher contrast sensitivity, visual clarity, and simple reaction time as compared to team sports players (i.e., basketball, soccer) that showed better spatial working memory. Interestingly, these visual skills were associated with the average number of goals or game points, strongly suggesting that performance might be directly correlated to the visual encoding during the match [49]. By means of the same Nike Station, baseball players were also screened for visual skills, searching for differences between professional hitters and pitchers; hitters showed the best depth perception and visual acuity [47]. As for hockey players, the visual skills of professional baseball players were positively associated with game statistics [50].

## 4. A Proposed Model of Visuomotor Integration

**Figure 3:**
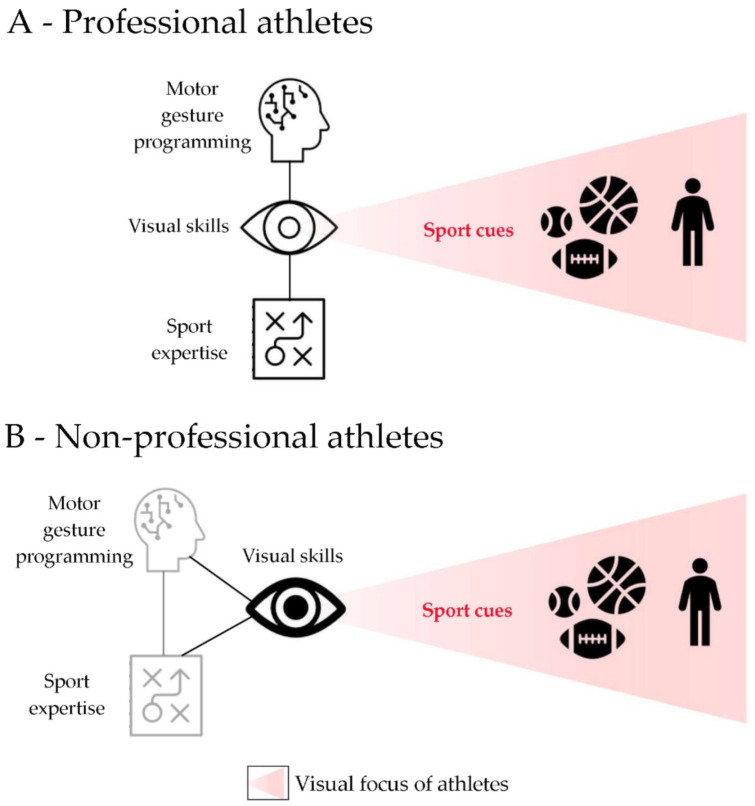
Proposed scheme of visuomotor integration model in professional panel (**A**) and non-professional panel (**B**) athletes. (**A**) Visual input and skills are integrated with motor control and sport expertise in professional athletes. (**B**) Visual abilities are the main component by which non-professionals compensate for the absence of sport experience in the programming of motor gestures. The blurred ray identifies the visual focus of athletes.

We approached this review with the hypothesis that visual skills could influence athletic performance as motor skills do. The literature certainly confirmed this insight, showing an impact on performance of varying degrees, mainly depending on the expertise of athletes and their training level [18,19,20,34,35]. According to this assumption, we propose a model of visuomotor integration that differs between professional and non-professional athletes: professionals optimally integrate visual skills, including stereoptic perception, with motor gesture programming and sport expertise, even if the visual input still remains a relevant afference [19,21] (Figure 3A); on the contrary, non-professionals encode action strongly relying on visual-dependent processes (Figure 3B) [25].

More specifically, the sport cues that could occur during a match (i.e., from the ball, opponents, and teammates’ movements) or related to a generic athletic performance are encoded by professional athletes by using visual skills, rapidly integrated with their experience, and processed at cortical level to generate an optimal motor response. On the other hand, non-professionals respond to sport cues by using mainly visual information rather than sport expertise, which they lack.

It is of note that, independently from the athletes’ level and as previously suggested by Basevitch et al. (2015) [37], visual input and skills are strictly related to motor gestures. Therefore, the training level of such visual abilities will impact performance of any disciplines and athletes.

## 5. Summary and Conclusions

The aim of this review was to put together what is known about the potential role of stereopsis in athletic performance. Although the area has not been exhaustively investigated yet, evidence indicates that stereopsis particularly affects the interceptive actions of ball sports, in which the trajectory and ball direction are finely encoded by the depth perception ability [15].

As for the trainability of visual skills, including stereopsis, it can be possible through sensory stations and light board training systems: improvements are reported in both professional and non-professional athletes, showing a positive correlation between the optimal visual encoding of the match (for example, in strategic sports) and game statistics [49,50]. A position-dependent improvement of visual skills has been also reported [41]. In interceptive sports, where catching and pitching are the most relevant skills, the training should be focused on object speed and trajectory, integrating muscular training with visual and visuomotor cues. In putting and shooting sports, training should be directed to the motor control with exercises emphasising proprioceptive and vestibular signals. In strategic sports, training should be focused on the peripheral vision, quick response time and decision-making skills, using exercises that active the stimulus-response system.

In the end, a vision training program appears relevant both in non-professional and professional athletes.

Differences emerged when comparing the visual abilities of athletes versus non-athletes [19,20,21]. In addition, a model of visuomotor integration during performance can be proposed according to the athletes’ experience level (professionals versus non-professionals); a vision-dependent approach characterises non-professionals, who compensate the lack of sport expertise by using mainly the visual input to encode the match (or performance). Thus, visual abilities should be trained starting from the early training sessions of novices. By contrast, professional athletes, for whom the visual input is, however, part of the information integration process, use the visual afference to a minor degree because they are able to program the motor gesture through proprioceptive and visual signals, sport and decision-making expertise [19,20,21,25]. In contrast to the muscolo-skeletal and cardiopulmonary/metabolic aspects of athletic training [51,52,53], studies on visual skills and stereopsis in sports [10,49] are still in their early stages, and there is a large potential for both building on scientific knowledge and technical development.

## Data Availability

The data presented in this study are available in this article.

## References

[1] Stine C.D., Arterburn M.R., Stern N.S. (1982). Vision and sports: A review of the literature. J. Am. Optom. Assoc..

[2] Hitzeman S., Beckerman S. (1993). What the literature says about sports vision. Optom. Clin. Off. Publ. Prentice Soc..

[3] Regan D., Gray R. (2001). Hitting what one wants to hit and missing what one wants to miss. Vis. Res..

[4] Sharma P. (2018). The pursuit of stereopsis. J. Am. Assoc. Pediatr. Ophthalmol. Strabismus.

[5] Newell A. (1994). Unified Theories of Cognition.

[6] McBeath M.K., Tang T.Y., Shaffer D.M. (2018). The geometry of consciousness. Conscious. Cogn..

[7] Lenoir M., Musch E., La Grange N. (1999). Ecological Relevance of Stereopsis in One-Handed Ball-Catching. Percept. Mot. Ski..

[8] Mazyn L.I.N., Lenoir M., Montagne G., Savelsbergh G.J.P. (2004). The contribution of stereo vision to one-handed catching. Exp. Brain Res..

[9] Mazyn L.I.N., Lenoir M., Montagne G., Delaey C., Savelsbergh G.J.P. (2007). Stereo vision enhances the learning of a catching skill. Exp. Brain Res..

[10] Burris K., Liu S., Appelbaum L. (2020). Visual-motor expertise in athletes: Insights from semiparametric modelling of 2317 athletes tested on the Nike SPARQ Sensory Station. J. Sports Sci..

[11] Boden L.M., Rosengren K.J., Martin D.F., Boden S.D. (2009). A comparison of static near stereo acuity in youth baseball/softball players and non–ball players. Optom.—J. Am. Optom. Assoc..

[12] Quintana M.S., Román I.R., Calvo A.L., Molinuevo J.S. (2007). Perceptual Visual Skills in Young Highly Skilled Basketball Players. Percept. Mot. Ski..

[13] O’Connor A.R., Tidbury L.P. (2018). Stereopsis: Are we assessing it in enough depth?. Clin. Exp. Optom..

[14] O’Connor A.R., Birch E.E., Anderson S., Draper H., FSOS Research Group (2010). The Functional Significance of Stereopsis. Investig. Opthalmol. Vis. Sci..

[15] Gao Y., Chen L., Yang S.-N., Wang H., Yao J., Dai Q., Chang S. (2015). Contributions of Visuo-oculomotor Abilities to Interceptive Skills in Sports. Optom. Vis. Sci..

[16] Regan D. (2012). Vision and cricket. Ophthalmic Physiol. Opt..

[17] Regan D. (1997). Visual factors in hitting and catching. J. Sports Sci..

[18] Müller S., Lalovic A., Dempsey A., Rosalie S., Harbaugh A.G. (2014). Pick-up of Early Visual Information to Guide Kinetics and Kinematics within a Group of Highly Skilled Baseball Batters. Percept. Mot. Ski..

[19] Hülsdünker T., Strüder H.K., Mierau A. (2018). The athletes’ visuomotor system—Cortical processes contributing to faster visuomotor reactions. Eur. J. Sport Sci..

[20] Laby D.M., Kirschen D.G., Govindarajulu U., Deland P. (2019). The Effect of Visual Function on the Batting Performance of Professional Baseball Players. Sci. Rep..

[21] Hülsdünker T., Strüder H.K., Mierau A. (2017). Visual Motion Processing Subserves Faster Visuomotor Reaction in Badminton Players. Med. Sci. Sports Exerc..

[22] Tanaka H., Iwami M. (2018). Estimating Putting Outcomes in Golf: Experts Have a Better Sense of Distance. Percept. Mot. Ski..

[23] Shim J., Chung H., Kim J. (2019). Directional aiming bias in golf putting. J. Sports Sci..

[24] Van Lier W.H., van der Kamp J., Savelsbergh G.J. (2011). Perception and Action in Golf Putting: Skill Differences Reflect Calibration. J. Sport Exerc. Psychol..

[25] Micarelli A., Viziano A., Lanzillotta A., Ruscello B., D’Ottavio S., Alessandrini M. (2019). Visual dependency and postural control on swing performance in golf players. Eur. J. Sport Sci..

[26] Kim Y., Chang T., Park I. (2019). Visual Scanning Behavior and Attention Strategies for Shooting among Expert Versus Collegiate Korean Archers. Percept. Mot. Ski..

[27] Mohammadi S.F., Amiri M.A., Naderifar H., Rakhshi E., Vakilian B., Ashrafi E., Behesht-Nejad A.-H. (2016). Vision Examination Protocol for Archery Athletes Along With an Introduction to Sports Vision. Asian J. Sports Med..

[28] Zhu X.-J., Li Y.-H., Liu L.-Q. (2019). Functional significance of stereopsis in professional table-tennis players. J. Sports Med. Phys. Fit..

[29] Natsuhara T., Kato T., Nakayama M., Yoshida T., Sasaki R., Matsutake T., Asai T. (2020). Decision-Making While Passing and Visual Search Strategy During Ball Receiving in Team Sport Play. Percept. Mot. Ski..

[30] Vera J., Jiménez R., Cárdenas D., Redondo B., García J.A. (2020). Visual function, performance, and processing of basketball players vs. sedentary individuals. J. Sport Health Sci..

[31] Jin P., Li X., Ma B., Guo H., Zhang Z., Mao L. (2020). Dynamic visual attention characteristics and their relationship to match performance in skilled basketball players. PeerJ.

[32] Stöckel T., Breslin G. (2013). The influence of visual contextual information on the emergence of the especial skill in basketball. J. Sport Exerc. Psychol..

[33] Czyż S., Kwon O.-S., Marzec J., Styrkowiec P., Breslin G. (2015). Visual uncertainty influences the extent of an especial skill. Hum. Mov. Sci..

[34] Williams A.M., Davids K., Burwitz L., Williams J.G. (1994). Visual Search Strategies in Experienced and Inexperienced Soccer Players. Res. Q. Exerc. Sport.

[35] Ando S., Kida N., Oda S. (2001). Central and Peripheral Visual Reaction Time of Soccer Players and Nonathletes. Percept. Mot. Ski..

[36] Pesce C., Tessitore A., Casella R., Pirritano M., Capranica L. (2007). Focusing of visual attention at rest and during physical exercise in soccer players. J. Sports Sci..

[37] Basevitch I., Tenenbaum G., Land W.M., Ward P. (2015). Visual and skill effects on soccer passing performance, kinematics, and outcome estimations. Front. Psychol..

[38] Paulus J., Tong J., Hornegger J., Schmidt M., Eskofier B., Michelson G. (2014). Extended stereopsis evaluation of professional and amateur soccer players and subjects without soccer background. Front. Psychol..

[39] Ghasemi A., Momeni M., Jafarzadehpur E., Rezaee M., Taheri H. (2011). Visual Skills Involved in Decision Making by Expert Referees. Percept. Mot. Ski..

[40] Piras A., Lobietti R., Squatrito S. (2014). Response Time, Visual Search Strategy, and Anticipatory Skills in Volleyball Players. J. Ophthalmol..

[41] Wimshurst Z.L., Sowden P.T., Cardinale M. (2012). Visual Skills and Playing Positions of Olympic Field Hockey Players. Percept. Mot. Ski..

[42] Rezaee M., Ghasemi A., Momeni M. (2012). Visual and Athletic Skills Training Enhance Sport Performance. Eur. J. Exp. Biol..

[43] Zwierko T., Puchalska-Niedbal L., Krzepota J., Markiewicz M., Woźniak J., Lubiński W. (2015). The Effects of Sports Vision Training on Binocular Vision Function in Female University Athletes. J. Hum. Kinet..

[44] Zimmerman A., Lust K.L., Bullimore M. (2011). Visual Acuity and Contrast Sensitivity Testing for Sports Vision. Eye Contact Lens Sci. Clin. Pract..

[45] Laby D.M., Kirschen D.G., Pantall P. (2011). The Visual Function of Olympic-Level Athletes—An Initial Report. Eye Contact Lens Sci. Clin. Pract..

[46] Clark J.F., Colosimo A., Ellis J.K., Mangine R., Bixenmann B., Hasselfeld K., Graman P., Elgendy H., Myer G., Divine J. (2015). Vision Training Methods for Sports Concussion Mitigation and Management. J. Vis. Exp..

[47] Schoemann M.D., Lochmann M., Paulus J., Michelson G. (2017). Repetitive dynamic stereo test improved processing time in young athletes. Restor. Neurol. Neurosci..

[48] Klemish D., Ramger B., Vittetoe K., Reiter J.P., Tokdar S.T., Appelbaum L.G. (2018). Visual abilities distinguish pitchers from hitters in professional baseball. J. Sports Sci..

[49] Poltavski D., Biberdorf D. (2015). The role of visual perception measures used in sports vision programmes in predicting actual game performance in Division I collegiate hockey players. J. Sports Sci..

[50] Burris K., Vittetoe K., Ramger B., Suresh S., Tokdar S.T., Reiter J.P., Appelbaum L.G. (2018). Sensorimotor abilities predict on-field performance in professional baseball. Sci. Rep..

[51] Ultimo S., Zauli G., Martelli A.M., Vitale M., McCubrey J.A., Capitani S., Neri L.M. (2018). Influence of physical exercise on microRNAs in skeletal muscle regeneration, aging and diseases. Oncotarget.

[52] Simioni C., Zauli G., Martelli A.M., Vitale M., Sacchetti G., Gonelli A., Neri L.M. (2018). Oxidative stress: Role of physical exercise and antioxidant nutraceuticals in adulthood and aging. Oncotarget.

[53] Masselli E., Pozzi G., Vaccarezza M., Mirandola P., Galli D., Vitale M., Carubbi C., Gobbi G. (2020). ROS in Platelet Biology: Functional Aspects and Methodological Insights. Int. J. Mol. Sci..

